# Acute, Chronic, and Everyday Physical Pain in Borderline Personality Disorder

**DOI:** 10.1007/s11920-024-01498-0

**Published:** 2024-04-10

**Authors:** Melissa Nance, Khrystyna Stetsiv, Ian A. McNamara, Ryan W. Carpenter, Johanna Hepp

**Affiliations:** 1 Department of Psychological Sciences, University of Missouri, St. Louis, St. Louis, USA; 2 Department of Psychosomatic Medicine and Psychotherapy, Medical Faculty Mannheim, Central Institute of Mental Health, Heidelberg University, J5, 68159 Mannheim, Germany

**Keywords:** Personality disorders, Borderline personality disorder, Physical pain, Chronic pain

## Abstract

**Purpose of Review:**

Physical pain is an underrecognized area of dysregulation among those with borderline personality disorder (BPD). Disturbances are observed within the experience of acute, chronic, and everyday physical pain experiences for people with BPD. We aimed to synthesize research findings on multiple areas of dysregulation in BPD in order to highlight potential mechanisms underlying the association between BPD and physical pain dysregulation.

**Recent Findings:**

Potential biological mechanisms include altered neural responses to painful stimuli within cognitive-affective regions of the brain, as well as potentially low basal levels of endogenous opioids. Emotion dysregulation broadly mediates dysregulation of physical pain. Certain psychological experiences may attenuate acute physical pain, such as dissociation, whereas others, such as negative affect, may exacerbate it. Social challenges between patients with BPD and healthcare providers may hinder appropriate treatment of chronic pain.

**Summary:**

Dysregulated physical pain is common in BPD and important in shaping health outcomes including elevated BPD symptoms, chronic pain conditions, and risk for problematic substance use.

## Introduction

Borderline personality disorder (BPD) is characterized by an enduring pattern of disturbances in affect, impulsivity, self-image, and interpersonal relationships [1]. Another, less recognized, area in which people with BPD commonly experience disturbances is physical health, especially the experience of physical pain [2–8]. As the body signals to the brain that something is awry, physical pain is both a ubiquitous experience and important for maintaining adaptive functioning. Dysregulation in the experience of pain, either in the direction of too little pain or too much, can have significant consequences. BPD has been associated with greater chronic and everyday pain and, in contrast, reduced sensitivity to acute pain. This apparent contradiction has been termed the “pain paradox” of BPD [9–11].

**Fig. 1:**
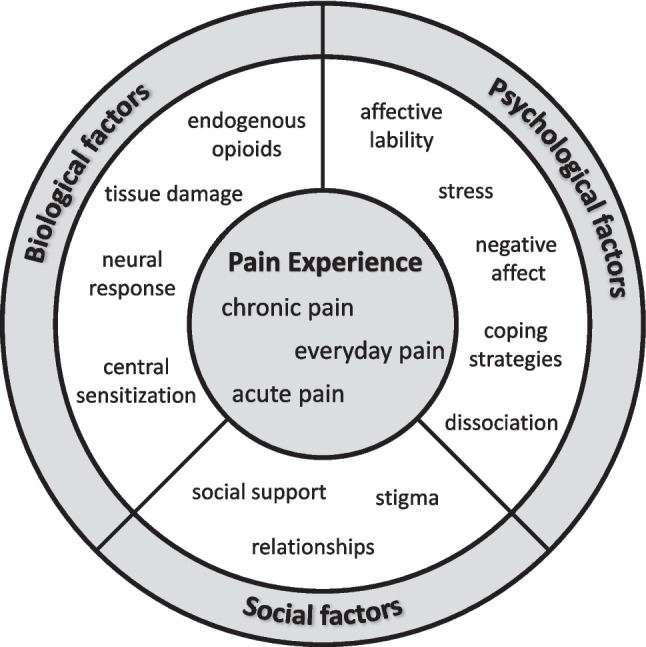
Biopsychosocial framework for understanding the relationship of physical pain and borderline personality disorder

In the current paper, we first define chronic, everyday, and acute pain and review the evidence for their relationship to BPD. Following this, we examine evidence for processes potentially related to pain dysregulation in BPD. In this review, we adopt a biopsychosocial framework (Fig. 1), which has been applied to both the experience of physical pain [12, 13] and BPD [14, 15] and may be particularly useful in highlighting mechanisms involved in their association. The biopsychosocial model of chronic pain conceptualizes the experience of pain as being shaped by biological (e.g., neural), psychological (e.g., affect), and social (e.g., relationships) mechanisms which interact with one another to influence pain experiences [12, 13]. Likewise, Linehan’s biosocial theory of BPD identifies interactions between biological, psychological, and social components [14]. We review evidence for the association of pain and BPD within each of these three interrelated domains.

Before proceeding, it is important to note that this review was initially conceptualized as a review of recent work on physical pain in personality disorders broadly. We conducted a comprehensive literature search of original studies listed in PubMed with the search terms “pain” OR “chronic pain” AND “personality disorder,” “paranoid,” “schizoid,” “schizotypal,” “antisocial,” “borderline,” “histrionic,” “narcissistic,” “avoidant,” “dependent,” “obsessive–compulsive,” “alternative model of personality disorders,” “HiTOP,” and “NSSI.” Studies on NSSI were further inspected to identify studies with samples that represented a personality disorder population. We considered studies that assessed categorical personality disorders and studies that assessed dimensional personality disorder traits within the past 5 years (January 1st, 2018–February 9th, 2023). The initial search yielded 582 results, from which 198 duplicate results were removed and 339 studies were removed for not assessing physical pain and personality disorders/traits. In total, *N* = 45 studies were identified that matched our criteria. Of the 45 articles identified, only 5 studies examined antisocial personality disorder/psychopathic traits, 1 study examined trait narcissism, and 2 studies examined dimensional personality traits. Articles focused on antisocial personality tended to examine empathy for other’s pain rather than one’s own experience of painful stimuli. We therefore focus this narrative review on pain in BPD. We return to this point, and the need for additional attention to the experience of pain in personality disorders broadly, in the conclusion.

### Chronic, Everyday, and Acute Pain

*Chronic pain* involves experiencing pain over time (typically ≥ 3 months) and is recognized as pain that exceeds normal healing time and lacks nociceptive benefits, such as functional warnings about injuries [16]. Chronic pain is prevalent in BPD samples and, in turn, BPD features are common in chronic pain samples. For instance, Heath et al. [17] found that 65% of patients entering outpatient treatment for BPD (*N* = 65) met DSM-IV criteria for pain disorder, a disorder characterized by pain that causes clinically significant distress or impairment where psychological factors are judged to have an important role in the onset, severity, exacerbation, or maintenance of the pain [18]. Likewise, a prior review suggests that approximately 30% of people with a chronic pain disorder meet criteria for BPD or report significant BPD traits [9] - a significant health disparity given the rate of BPD among the general population of the United States (US) is around 2% [19, 20]. Physical pain, particularly chronic pain, can lead to a reduction in participation in daily activities, which we will refer to as pain-related disability.

Aside from chronic pain, virtually everyone experiences *everyday pains* that are context-specific, relatively short-lived, and can be generally self-managed (e.g., with over-the-counter pain medication). As many as 80–89% of those with BPD report experiencing some level of physical pain within the past day [17, 21]. Within a non-clinical sample (*N* = 206), BPD features were cross-sectionally associated with greater past-year nonchronic pain [11]. Furthermore, in an ambulatory assessment study, Carpenter et al. [22••] observed that BPD outpatients (*n* = 26) reported greater everyday pain intensity and variability than community participants (*n* = 26). These findings suggest that people with BPD are prone to experiencing pain in their daily lives, including pain that does not rise to the definition of chronic pain. These everyday pains are worthy of attention. Similar to mood dysregulation, daily experience of pain dysregulation can potentially lead to significant impacts on functioning [23]. It may also contribute to why people with BPD appear to be over-represented in samples of people seeking pain treatment [9] and may lead them to self-manage their pain in maladaptive ways (e.g., substance use [24]).

Perception of *acute pain* in BPD has received considerable attention because people with BPD frequently engage in non-suicidal self-injury (NSSI; i.e., tissue damage without the intent to die), an inherently painful behavior. Experimental work has found that people with BPD and a history of NSSI report reduced acute pain in response to painful stimuli. For example, Fales and colleagues [25••] recently reviewed laboratory pain induction studies that examined acute pain perception of individuals with BPD. Overall, those with BPD showed a higher pain threshold (the amount of time elapsed until pain onset after a stimulus is applied) and lower pain intensity (how painful a stimulus is perceived) relative to comparison participants. In contrast to this experimental work, however, a recent large (*N* = 1630) retrospective study of people with a history of NSSI (almost all of whom reported significant BPD features) found that most people who self-harm report mild-to-moderate pain during NSSI, and BPD features were not associated with amount of pain reported [26]. Thus, there appears to be a discrepancy between what people report in the laboratory in response to standardized stimuli and what they experience in their daily lives. It may be that people with BPD do have generally greater acute pain tolerance, but also that pain plays an important role in experiencing benefits from NSSI (e.g., reduction of negative affect). These benefits may lead them to self-harm in a manner that increases their experience of pain [26, 27]. A significant challenge, however, of understanding the relationship of BPD and acute pain experience is distinguishing possible effects of BPD from those of having a history of NSSI, given the considerable overlap of BPD and NSSI history.

## Biological Processes

Pain arises when nociceptive nerves are stimulated in the body; the resulting signal is transmitted up the spinal cord to the brain, and the signal is then interpreted in the brain. At times, the central nervous system can incorrectly interpret stimuli as painful due to an increased responsiveness of nociceptors to sub-threshold input, a phenomenon known as central sensitization. Central sensitization may be a relevant process for understanding the tendency for people with BPD to report more chronic and everyday pain. In a sample of chronic pain patients (*N* = 181), those with BPD were more likely to meet criteria for fibromyalgia, which is the prototypical central sensitization disorder [28•]. Furthermore, those with a chronic pain disorder and greater BPD features experienced greater poly-somatic complaints associated with central sensitization than those with chronic pain disorder but without elevated BPD features [28•]. These findings indicate that chronic pain experiences for people with BPD could be, in part, related to the perception or appraisal of sensory inputs as painful that other individuals would not perceive as such.

Atypical perception of innocuous stimuli in BPD has been demonstrated through recent laboratory work. For instance, people with BPD (*n* = 25) exposed to pleasant touch stimuli (e.g., a brush stroke on the hand) rated it as less pleasant than control participants (*n* = 25) [29•], which could be related to negative evaluation tendencies seen in BPD. Individuals with BPD have also shown differences in threshold and perceived intensity for innocuous stimuli that align with prior research on painful stimuli. Recent work found that those with BPD report lower intensity of pleasant touch [29•] as well as higher thresholds to detect warm thermal stimulation (*n* = 22 patients with BPD compared to *n* = 33 control participants) [30•]. These results suggest those with BPD may have hyposensitivity not only to painful laboratory stimuli, but also to innocuous stimuli. This, however, is the opposite of what would be expected if BPD was associated with central sensitization. It is worth noting that these findings deviate from previous research that did not find differences in the ability to detect non-painful stimuli for those with BPD [31, 32], indicating a need for continued research on the perception of non-painful stimuli in BPD and factors, such as body mass [32], that may contribute to hyposensitivity. It is also possible that the effect of BPD on perception of innocuous stimuli is only large enough to be detected when individuals with current BPD are compared to individuals with no prior psychopathology, given that the two studies that did not find differences for individuals with BPD (*n* = 27 and *n* = 29) included individuals who had a history of major depressive disorder with no current psychopathology (*n* = 20), and healthy controls (*n* = 44), or individuals with remitted BPD (*n* = 19) and healthy controls (*n* = 22).

Multiple brain areas are involved in pain processing, including affective, cognitive, and somatosensory areas, reflecting the complex integration of sensory and emotional experiences that culminate in the perception of pain. When unpleasant, but non-painful, electric stimulation was used in a recent fMRI study, no group differences were observed between control participants (*n* = 15) and women with BPD (*n* = 15) in the right primary somatosensory cortex, right secondary somatosensory cortex, posterior insula, right posterior midcingulate cortex, and right supplementary motor area [33]. A separate fMRI study found that individuals with a history of NSSI (*N* = 81, ~ 1/3 of whom reported having BPD) showed greater neural activation in the primary and secondary somatosensory cortex during thermal stimulation than control participants, which may be explained by the fact that their fMRI protocol used individually calibrated heat pain and the NSSI group required higher temperatures to detect pain [34]. These studies suggest that individuals with BPD respond to pain and innocuous stimuli within somatosensory areas in ways similar to those without BPD; however, cognitive and affective neural processing of painful stimuli may explain differences in the experience of pain. For instance, a recent review of 13 fMRI studies investigating BPD and self-injurious and suicidal behaviors suggests that people with BPD and a history of self-harm and/or suicide attempts show altered brain activity in regions involved in cognitive processes and affect regulation. Specifically, their review found evidence for altered neural patterns for individuals with BPD in the lateral orbitofrontal cortex, dorsal anterior cingulate cortex, and medial and dorsolateral prefrontal cortex, as well as in the nucleus accumbens and amygdala in response to pain stimulation [35].

Reduced sensitivity to acute pain could increase risk for engaging in NSSI by removing a natural physiological barrier to self-harm [36]. At the same time, the experience of physical pain may actually be important for experiencing desired effects of NSSI, such as emotional relief. This may occur either via pain itself (pain onset) or the reduction of pain following self-harm (pain offset) [26, 37]. Olié et al. [38] recently found heightened activation in the nucleus accumbens in people with BPD (*n* = 20) compared to controls (*n* = 23) following thermal stimulation, potentially indicating a rewarding effect of pain experience. These fMRI findings suggest that people with BPD might experience a pleasant sensation in response to heat that others perceive as painful, underscoring the affective processing involved in pain perception. A recent study which recruited individuals with BPD with and without history of NSSI, alongside control participants (*n* = 20 in each group), found that those with BPD and history of NSSI showed heightened responses in the left lateral orbitofrontal cortex in response to unexpected rewards, which may indicate a propensity to learning that NSSI can provide a rewarding effect [39].

Recent work has also investigated the role of the endogenous opioid system in how people with BPD experience pain. Endogenous opioids are produced in the brain and circulate widely throughout the nervous system. Opioids are important for maintaining the body’s homeostasis generally and play a specific role in regulating the perception of pain by reducing acute pain [40]. That the release of endogenous opioids reduces acute pain levels in humans generally is well-established. More recently, certain symptoms of BPD, such as dysphoria and risk-taking behaviors, have been suggested as indicators of low baseline levels of endogenous opioids [41]. Supporting this idea, opioid antagonist treatments have recently been shown to reduce BPD symptoms in a retrospective chart analysis of individuals with BPD (*N* = 161) who received inpatient treatment [42]. Opioid antagonists may acutely block the rewarding effects of problematic coping behaviors that would otherwise activate the endogenous opioid system (e.g., NSSI, substance use), while chronic administration may restore balance in the neurotransmission of endogenous opioids. However, evidence for the use of opioid antagonists to treat BPD is preliminary and more research is needed on the mechanisms behind improvement in symptoms.

Stanley and colleagues [43] first proposed that people who engage in NSSI might have an endogenous opioid deficiency. This was based on findings that those with a cluster B personality disorder and history of self-injurious behavior (*n* = 14) had lower levels of cerebrospinal β-endorphin and met-enkephalin compared to a diagnostically matched group of individuals with no history of self-harm (*n* = 15) [43]. Specifically, they posited that genetic vulnerability and early life adversity could lead to chronically low levels of endogenous opioids, which individuals attempt to counter by engaging in self-injurious behaviors to stimulate release of endogenous opioids. A recent study found that adolescents with a history of NSSI (*n* = 94) showed lower plasma β-endorphin levels, increased pain thresholds, and lower pain intensity compared to control participants (*n* = 35) [44]. Furthermore, in a recent ambulatory assessment study in individuals with DSM-5 NSSI disorder (*N* = 51, 63% meeting criteria for BPD), Störkel et al. [45] observed that NSSI acutely increased salivary β-endorphin levels and found evidence of a positive association between severity of the self-inflicted injury and β-endorphin levels. While these studies focus on NSSI, the majority of participants met for BPD and it is possible biological mechanisms and hypothesized causes (stress and trauma exposure) are applicable to people with BPD irrespective of NSSI history, although more work is needed to understand whether this is the case.

Low levels of circulating endogenous opioids might also leave people with BPD at risk of experiencing chronic pain and more intense everyday pain. Considerable evidence has accumulated for endogenous opioid dysfunction in chronic pain, particularly low endogenous opioid CSF/plasma levels in chronic pain patients [46, 47]. Among chronic pain patients (*N* = 28), endogenous opioid system dysfunction has been associated with elevated acute and chronic pain sensitivity, as well as pain-related disability [48]. Limited evidence suggests that individuals with BPD may have low baseline levels of endogenous opioids. Prossin et al. [49] used positron emission tomography to examine availability of β-endorphin receptors in women with BPD (*n* = 18) and matched comparison participants (*n* = 14). The authors found that BPD was associated with greater baseline β-endorphin receptor availability in the orbitofrontal cortex, caudate, nucleus accumbens, and amygdala, and further observed differences in receptor binding potential in response to sadness in the pregenual anterior cingulate, left orbitofrontal cortex, left ventral pallidum, left amygdala, and left inferior temporal cortex. Findings from this study have been interpreted as evidence of dysregulation in the endogenous opioid system for individuals with BPD, including low basal levels of endogenous opioids and compensatory responses in opioid receptors within reward processing regions. In addition to chronic pain outcomes, risk for problematic opioid use seen in BPD populations may be linked to endogenous opioid system dysfunction [50•].

## Psychological Processes

Emotion dysregulation is a core component of BPD, conceptualized as including heightened and labile negative affect, emotion sensitivity, and maladaptive coping strategies [51]. Negative affect has been associated with pain experiences among those with BPD. For example, Carpenter et al. [22••] found, in their ecological momentary assessment study, that everyday physical aches and pains were associated with negative affect for both BPD (*n* = 26) and community (*n* = 26) individuals, but that pain predicted future negative affect more strongly in the BPD group. Reynolds et al. [52] used path models in a cross-sectional chronic pain sample (*N* = 147) to examine whether trait affective lability, anxiety, and depression accounted for associations between BPD features and multiple indices of pain over the past year (pain severity, pain-related affective interference, and pain-related activity interference). Pain severity measured pain on an 11-point scale at its worst, least, and average over the past year, as well as current level of pain. Pain-related activity interference assessed pain’s interference with general activity, walking ability, and normal work whereas affective interference assessed interference with mood, interpersonal relationships, and life enjoyment. Although their study design did not allow for examining causal mediation, they found that affective lability and trait anxiety showed significant indirect effects accounting for the association between BPD features and all three indices of pain and pain interference. Additionally, serial path models indicated that anxiety around pain experience accounted for additional variance in the model [52]. Similarly, Johnson et al. [28•] assessed negative affect and BPD symptoms cross-sectionally within a chronic pain sample (*N* = 181) and found that associations between BPD features and chronic pain symptoms were accounted for by level of anxiety and depression. Negative affect has also been shown to mediate the relationship between interpersonal stressors and health problems across multiple domains (i.e., headaches, stomachaches, and muscle pains) in daily life for individuals with BPD (*n* = 81) and depression (*n* = 50) [53]. Together, these findings indicate that emotion dysregulation may contribute to the high prevalence of chronic and everyday pain observed among individuals with BPD. At the same time, the direction of this association remains unclear, as it is also possible that regularly experiencing pain worsens emotion dysregulation over time.

Maladaptive coping strategies contribute to emotion dysregulation within BPD and may also contribute to pain dysregulation by increasing negative emotions and emotional lability over time. The use of NSSI to regulate negative affect is common in BPD and while it may have short-term benefits, it may ultimately increase dysregulation. Use of emotion suppression, another potentially maladaptive coping strategy, in response to social stressors and under high distress has recently been associated with greater daily pain interference for individuals with BPD features (*N* = 89), even after accounting for daily pain intensity [54].

In addition to finding generally higher pain threshold and lower pain intensity among those with BPD, Fales and colleagues’ review [25••] indicated that BPD participants had higher pain tolerance (the length of time one is able to endure a painful stimulus) specifically under conditions of emotional distress. This process, known as stress-induced analgesia, has also been observed in other clinical and non-clinical groups, as well as in animals, and is believed to be a generally adaptive, evolved pain suppression response [55]. However, outside of the laboratory, evidence for stress-induced analgesia during NSSI is lacking. For instance, in a recent large (*N* = 1630) cross-sectional study of people with a history of NSSI who were high in BPD features, greater emotional distress before NSSI predicted greater NSSI pain [26]. It remains unclear whether and why stress-induced analgesia may be enhanced in individuals with BPD and what this means for people with BPD [27].

Dissociation, a psychological state characterized by perceived disconnection, is another psychological process that may impact the experience of acute pain in BPD. Transient dissociation is common among people with BPD, particularly in response to stress. Furthermore, prior review of 70 studies indicates that BPD symptom severity is associated with both experiencing dissociation and decreased pain perception [56]. A recent study by Chung et al. [57•] found that individuals who currently met criteria for BPD (*n* = 25) showed greater pain hyposensitivity during experimental pain induction than those with remitted BPD (*n* = 20) and a non-clinical control group (*n* = 24). This study also examined pain perception and dissociation following a personalized stress task, finding that stress and dissociation independently contributed to pain hyposensitivity for both the current and the remitted BPD groups [57•]. Dissociation has also been associated with lower self-reported NSSI pain [26]. Broadly, there is evidence for adverse effects of dissociation on affective-cognitive functioning, body perception, and treatment response in BPD [58]. Dissociation has also been suggested as a transdiagnostic symptom involved in the pathway between adverse childhood experiences, psychiatric disorders (e.g., BPD and post-traumatic stress disorder), and pain chronicity or pain-related disability [59].

Dissociation commonly includes a distorted perception of the self, one’s body, or one’s sense of agency. A factor that may be related to experiencing dissociation is a person’s ability to sense their own body, or their interoceptive sensitivity. Individuals with high interoceptive sensitivity are better able to differentiate between various signals from their body’s organs (e.g., heart rate). Among those with BPD, interoceptive sensitivity has been associated with pain intensity ratings in laboratory pain tasks. Specifically, low body awareness has been associated with analgesia [60], which may facilitate NSSI behaviors and explain prior findings of high pain threshold and low pain intensity among those with BPD. However, people with higher levels of interoceptive sensitivity and BPD (*n* = 46) rated lower temperatures as evoking moderate pain and reported higher pain intensity ratings than control participants (*n* = 47) [60]. The fact that the effect of interoceptive sensitivity was observed for those with BPD but not control participants may be explained if individuals with BPD specifically respond to internal stimuli with anxiety and catastrophic thinking, such as in response to recurrent pain.

## Social Processes

Social processes that affect acute and chronic pain in BPD play out in different contexts, which include not only the context of personal relationships but also the healthcare context. Within personal relationships, the BPD feature of negative relationships (i.e., unstable interpersonal relationships) has been associated with increased rates of pain-related disability, general disability, and unemployment in a chronic pain sample (*N* = 147) [61••]. Individuals with maladaptive interpersonal patterns may experience a lack of social support, as well as more conflictual relationships with healthcare providers, and discontinuity in healthcare providers as a result of conflict, each of which could contribute to greater chronic pain and disability. People with BPD utilize healthcare services at rates greater than the general population [62], and yet, many who seek treatment for chronic pain experience barriers to accessing treatment, which again underlines the need for scientific attention. Chronic pain is a subjective experience and medical providers must rely on patient reports of their pain experiences. However, patients with BPD may be perceived as untrustworthy by healthcare providers, even when their diagnosis is not known. When raters in three samples (*N* = 98, *N* = 94, *N* = 44) viewed video samples of BPD and healthy control participants as targets, they rated BPD targets as less likeable, less trustworthy, and less cooperative [63, 64]. Providers may perceive patients with BPD as less trustworthy because they report high levels of pain severity and express frustration with the (lack of) treatment provided for their pain conditions [65•] or as a result of negative stigma [66]. The interpersonal deficits seen in BPD, and those in chronic pain samples with BPD features, may contribute to the perpetuation of chronic pain experiences in spite of attempts to receive treatment for chronic pain or contributory medical conditions.

Appropriate treatment for chronic pain is an especially important issue for those with BPD given challenges specific to this population. Despite concerns about risks, prescription opioids remain a primary treatment option of chronic pain, particularly in the US [67]. Frankenburg et al. [68] found that participants with BPD (*n* = 264) were more likely to report prescription opioid use than comparison participants with another personality disorder (*n* = 63), which may be due to higher rates of co-occurrence of BPD with chronic pain conditions. Additional evidence suggests that people with BPD may be at risk for problematic prescription opioid use. For instance, in a sample of outpatients who currently or have previously been prescribed pain medication (*N* = 185), BPD symptoms were associated with measures of prescription pain medication misuse [69]. In college student samples, BPD features have been associated with negative reinforcement motives (coping and pain) for prescription opioid use (*N* = 594) [70] and measures of misuse, including opioid consequences and dependence features (*N* = 606) [71]. Finally, in samples in treatment for chronic pain (*N* = 147) and samples in treatment for substance use disorder (*N* = 208), BPD features have been associated with prescription opioid misuse, with particularly strong associations between measures of misuse and the identity disturbance and self-harm/impulsivity facets of BPD [72•, 73]. It is important to note that these findings do not indicate that people with BPD cannot benefit from appropriate opioid therapy. However, patients with BPD and chronic pain may benefit from additional monitoring and resources (e.g., support, psychoeducation, psychosocial therapy) when completing a course of prescription opioids.

## Conclusions

We reviewed evidence on chronic, acute, and everyday pain in BPD from the past 5 years, and conclude that BPD symptom severity is positively associated with pain dysregulation and pain-related disability [28•, 52, 61••]. The experiences of pain in BPD may be mediated by interconnected biopsychosocial processes, manifesting in emotion dysregulation and dysregulated experiences of physical pain. Recent fMRI studies suggest that neural activation in cognitive/affective areas in people with BPD differs from healthy control participants [35], but those with BPD generally showed no differences from control participants in somatosensory areas [33, 34]. While preliminary and based on small sample sizes, these fMRI studies may indicate that altered pain perception is specifically related to affective processing of sensory information. This idea is reinforced by recent findings that negative affect and affective instability contribute to chronic pain experiences in individuals high in BPD features [28•, 52]. There is also evidence to suggest that pain and emotion dysregulation may be manifestations of chronically low endogenous opioids, which regulate the experience of pain [41–43, 49, 50•]. Social challenges between providers and patients with BPD features [63, 64, 65•, 66] could lead to non-pharmacological treatments for chronic pain being underutilized in BPD populations. Providers may also be less willing to prescribe opioids to people with BPD, potentially leading people with BPD to go without adequate pain treatment or to seek out non-prescribed opioids or other substances to self-medicate their pain.

In summary, our review indicates that dysregulated physical pain is common in BPD and important in shaping health outcomes, including risk for opioid misuse, BPD symptoms, and chronic pain. Evidence suggests that emotion dysregulation components (emotion sensitivity, negative affect, affective lability, and maladaptive coping behaviors) mediate the relationship between BPD and dysregulated physical pain. Underlying emotion dysregulation, differences in the cognitive and affective neural processing of information, as well as physiological deficits in the endogenous opioids system, likely also contribute to physical pain dysregulation. Mistrust and interpersonal challenges between patients and providers may exacerbate chronic pain symptoms and disability levels by hindering appropriate treatment of chronic pain.

We recommend regularly assessing BPD traits in chronic pain populations and vice versa, and encourage comprehensive care for physical and mental health. Additionally, chronic pain should be assessed as a psychotherapy outcome for BPD populations, along with measures of BPD symptoms, emotion dysregulation, and negative affect. Evaluating multiple indices of physical and mental health regularly in this population can provide evidence for directionality and indicate if improvements in emotion regulation improve pain experiences.

Beyond BPD, pain processes in other categorical personality disorders and the new dimensional personality disorder diagnosis in ICD-11 warrant further attention [74]. The present article focused on BPD because this was the disorder commonly examined in recent literature and it is also the personality disorder with the highest reported incidence of chronic pain [21] and largest body of evidence on dysregulated acute pain [25••]. However, there is evidence for high rates of trauma history and emotion dysregulation in other personality disorders [75]*,* which may similarly alter the endogenous opioid system [76] and neural responses to pain, contributing to altered pain perception. Furthermore, BPD has been suggested to reflect general personality pathology [77]. Biological, psychological, and social processes observed within BPD samples could occur on a transdiagnostic and dimensional spectrum, but research on dimensional models of personality disorder and pain was exceedingly rare within the past five years.
